# TCRαβ-Depleted Haploidentical Grafts Are a Safe Alternative to HLA-Matched Unrelated Donor Stem Cell Transplants for Infants with Severe Combined Immunodeficiency

**DOI:** 10.1007/s10875-022-01239-z

**Published:** 2022-03-19

**Authors:** Christo Tsilifis, Su Han Lum, Zohreh Nademi, Sophie Hambleton, Terence J. Flood, Eleri J. Williams, Stephen Owens, Mario Abinun, Andrew J. Cant, Mary A. Slatter, Andrew R. Gennery

**Affiliations:** 1 Paediatric Haematopoietic Stem Cell Transplant Unit, Great North Children’s Hospital (GNCH), Clinical Resource Building, Royal Victoria Infirmary, Victoria WingFloor 4, Block 2, Queen Victoria Road, Newcastle upon Tyne, NE1 4LP UK; 2 Translational and Clinical Research Institute, Faculty of Medical Sciences, Newcastle University, Newcastle upon Tyne, NE2 4HH UK

**Keywords:** SCID, Haploidentical, TCRαβ, T-cell depletion, Viremia, GvHD, Conditioning

## Abstract

Hematopoietic stem cell transplantation and gene therapy are the only curative therapies for severe combined immunodeficiency (SCID). In patients lacking a matched donor, TCRαβ/CD19-depleted haploidentical family donor transplant (TCRαβ-HaploSCT) is a promising strategy. Conditioned transplant in SCID correlates to better myeloid chimerism and reduced immunoglobulin dependency. We studied transplant outcome in SCID infants according to donor type, specifically TCRαβ-HaploSCT, and conditioning, through retrospective cohort analysis of 52 consecutive infants with SCID transplanted between 2013 and 2020. Median age at transplant was 5.1 months (range, 0.8–16.6). Donors were TCRαβ-HaploSCT (*n* = 16, 31.4%), matched family donor (MFD, *n* = 15, 29.4%), matched unrelated donor (MUD, *n* = 9, 17.6%), and matched unrelated cord blood (CB, *n* = 11, 21.6%). Forty-one (80%) received fludarabine/treosulfan-based conditioning, 3 (6%) had alemtuzumab only, and 7 (14%) received unconditioned infusions. For conditioned transplants (*n* = 41), 3-year overall survival was 91% (95% confidence interval, 52–99%) for TCRαβ-HaploSCT, 80% (41–98%) for MFD, 87% (36–98%) for MUD, and 89% (43–98%) for CB (*p* = 0.89). Cumulative incidence of grade II–IV acute graft-versus-host disease was 11% (2–79%) after TCRαβ-HaploSCT, 0 after MFD, 29% (7–100%) after MUD, and 11% (2–79%) after CB (*p* = 0.10). 9/10 patients who received alemtuzumab-only or unconditioned transplants survived. Myeloid chimerism was higher following conditioning (median 47%, range 0–100%) versus unconditioned transplant (median 3%, 0–9%) (*p* < 0.001), as was the proportion of immunoglobulin-free long-term survivors (*n* = 29/36, 81% vs *n* = 4/9, 54%) (*p* < 0.001). TCRαβ-HaploSCT has comparable outcome to MUD and is a promising alternative donor strategy for infants with SCID lacking MFD. This study confirms that conditioned transplant offers better myeloid chimerism and immunoglobulin freedom in long-term survivors.

**Supplementary Information:**

The online version contains supplementary material available at 10.1007/s10875-022-01239-z.

## Introduction

Severe combined immunodeficiency (SCID) is a pediatric emergency, and definitive treatment by hematopoietic stem cell transplantation (HSCT) or gene therapy should be performed promptly. This urgency is well-founded, as age > 3.5 months at HSCT confers higher mortality risk compared to those aged <3.5 months, with active or resolved infection further compounding this risk [1–3].

While HLA-matched siblings remain the first donor of choice [1, 2, 4], < 25% of patients have a matched sibling or family donor (MSD, MFD) available, necessitating consideration of alternative donors such as matched unrelated donors (MUD), unrelated cord blood (CB), or mismatched family donors. MUD searches introduce delay in time-to-transplant, increasing the risk of infection-related morbidity and mortality, while use of a cord graft introduces risk of either slower neutrophil and lymphocyte reconstitution if serotherapy is used [5], or enhanced rates of graft-versus-host disease (GvHD) if serotherapy is omitted [6], and precludes harvesting additional cells for various therapies. An alternative is using a mismatched family donor, with ex vivo T-lymphocyte depletion to reduce alloreactivity; while prompt availability of a parental donor may favor their use, this must be balanced against the risks of acute GvHD and delayed T-lymphocyte reconstitution, particularly in the context of viremia [7]. CD3 + TCRαβ/CD19-lymphocyte depletion is increasingly used in SCID and non-SCID inborn errors of immunity (IEI) HSCT [8–10] due to increased overall survival and reduced rates of GvHD compared to previous strategies such as CD34 + selection [11], though data on its use in SCID are sparse.

Previous recommendations for conditioning pre-HSCT for SCID vary by subtype and clinical status; the latest European Societies for Immunodeficiencies and Bone Marrow Transplantation (ESID/EBMT) guidance recommends conditioning, for improved rates of myeloid engraftment and immunoglobulin independence [12]. We explored the outcomes of HSCT after TCRαβ/CD19-depleted haploidentical donor (TCRαβ-HaploSCT) compared to other donor types, and the impact of conditioning.

## Methods

A retrospective cohort analysis of 52 consecutive patients undergoing first HSCT for SCID between 2013 and 2020 was performed. One patient was excluded (failed lentiviral gene therapy for adenosine deaminase [ADA] deficient-SCID leading to pancytopenia and monosomy 7, followed by successful HSCT). In the study, conditioned transplant included patients who received cytoreductive chemotherapy and serotherapy while unconditioned transplant referred to patients who did not receive any chemotherapy or had alemtuzumab only. Clinical and laboratory data were retrieved from the transplantation database, patients’ medical files, and laboratory records. Written informed consent was obtained from the patients and/or parents or legal guardians of the patients as per institutional practice for HSCT.

All patients underwent surveillance for cytomegalovirus (CMV), adenovirus, Epstein Barr virus (EBV), human herpes virus type 6 (HHV6) viremia, and respiratory and gut viruses performed weekly. All patients received antimicrobial prophylaxis against fungi, *Pneumocystis jiroveci* (PCP), and human herpesvirus reactivation. All patients received immunoglobulin replacement until normal IgM levels were evident. Enumeration of CD3 + , CD4 + , CD8 + , CD19 + , CD16/56 + , CD4 + CD45RA + , CD4-CD45RA + , and activated lymphocytes (denoted by HLA-DR +) by flow cytometry was performed pre-HSCT, and at months 1, 2, 3, 4, 5, 6, and 12 post-HSCT, and at latest follow-up. Donor chimerism analysis results were recorded according to whole blood and specific lineage, where available, at months 1, 3, 6, and 12 post-HSCT and at latest follow-up. Donor hematopoietic chimerism was monitored by molecular techniques.

The primary endpoints were overall survival (OS) and GvHD-free, event-free survival (GEFS). GEFS was defined as survival without graft failure, second procedure, grade III–IV acute GvHD (aGvHD), and chronic GvHD (cGvHD). Secondary endpoints were aGvHD, cGvHD, toxicities, and viremia. In the statistical analysis, quantitative variables were described with median and range while categorical variables were reported with counts and percentages. The association between continuous variables was assessed with the use of Wilcoxon rank-sum test when comparing two groups and Kruskal–Wallis test when comparing more than two groups. Subgroup differences in OS and GEFS were evaluated by log-rank test. Competing risks methods were used for the cumulative incidence of acute and chronic GVHD, with competing events death. Subgroup differences in cGvHD and aGvHD were evaluated by Gray’s test. All estimates are reported with 95% confidence intervals. All *p*-values quoted are two-sided, with a level of significance of 0.05. Statistical analyses were performed using STATA 14.2 and were generated with GraphPad Prism.

## Results

Of 51 patients, 48 had genetically or biochemically confirmed etiologies for SCID, 2 had T_low_B + NK + SCID, and one presented with an Omenn-like syndrome (Supplemental Table S1). Fourteen (27.4%) patients had a newborn diagnosis of SCID due to previous family history. Median age at diagnosis was 2.1 months (range: 0–21.8). The median age at transplant was 6.1 months (1.0–16.6) for non-ADA SCID and 4.0 (0.8–43.3) for ADA SCID (*p* = 0.42). Interval to transplant was significantly shorter in TCRαβ-HaploSCT (median 2.3 months, range 0.6–9.8 months) compared to adult MUD recipients (median: 3.9 months, range 2.0–14.6 months) (*p* = 0.039); this analysis excluded patients with ADA-SCID, who were treated with pegylated-ADA while awaiting MUD search, if MFD were unavailable.

There were no statistically significant differences between TCRαβ-HaploSCT and T-replete groups in pre-transplant comorbidities, infection history, or active viremia at HSCT (Supplemental Table S1).

Patients received grafts from either TCRαβ/CD19-depleted haploidentical parental donors (*n* = 16) or T-replete MFD (*n* = 15, of which 10 were siblings; 10 marrow, 5 PBSC), adult MUD (*n* = 9, 3 marrow, 6 PBSC), or unrelated cord blood (CB, *n* = 11) donors. Forty-one (80%) patients received treosulfan/fludarabine-based conditioning, 3 (5.9%) patients received alemtuzumab serotherapy only, and 8 (15.7%) patients received unconditioned infusions. Graft composition between donor types varied, with significantly higher median total nucleated cell and CD34 + cell doses in TCRαβ-HaploSCT (*p* < 0.001, **Table ****1**). The median TCRαβ cell dose was 4.50 (range: 1.2–20.0) × 10^4^ cells/kg, with 10 (62.5%) receiving < 5 × 10^4^ cells/kg.

**Table 1:** Patient pre-HSCT and graft and transplant characteristics, and post-HSCT complications, infection status, and outcome

		TCRαβ/CD19-depleted haploidentical donor (*n* = 16)		HLA-matched T-replete graft						*p*-value
				Family donor (*n* = 15)		Adult unrelated donor (*n* = 9)		Cord blood donor (*n* = 11)		
**Pre-HSCT characteristics**										
Median age at diagnosis, months		3.3	(0–12.7)	1.3	(0–8.8)	2.3	(0–9.4)	2.1	(0–13.8)	0.535
Median age at transplant, months		6.2	(1.7–16.4)	4.3	(1.0–10.2)	8.5	(2.2–16.6)	7.0	(1.7–15.6)	0.233
Median interval from diagnosis to transplant, months**		2.3	(0.6–9.8)	1.6	(1.0–3.2)	3.9	(2.0–14.6)	1.6	(0.3–2.3)	0.039*
**Transplant characteristics**										
Stem cell source	Cord blood	-	-	-	-	-	-	11	(100.0%)	< 0.001*
	Marrow	-	-	10	(66.7%)	3	(33.3%)	-	-	
	PBSC	16	(100.0%)	5	(33.3%)	6	(66.7%)	-	-	
Median TNC dose, × 10^8^ cells/kg		16.5	(1.0–96.0)	9.0	(3.1–21.0)	12.9	(7.1–20.4)	2.0	(0.9–3.4)	< 0.001*
Median CD34 + dose, × 10^6^ cells/kg		24.0	(0.8–60.9)	9.9	(3.0–25.7)	12.8	(4.6–24.2)	0.8	(0.2–1.5)	< 0.001*
Median CD3 + dose, × 10^8^ cells/kg		0.16	(0.02–5.60)	1.40	(0.38–7.70)	4.30	(0.25–5.60)	0.48	(0.11–4.70)	< 0.001*
Median CD19 + dose, × 10^7^ cells/kg		0.01	(0–1.80)	5.60	(1.90–18.00)	7.90	(1.10–23.00)	1.30	(0.21–11.00)	< 0.001*
Median TCRαβ + dose, × 10^4^ cells/kg		4.50	(1.20–20.00)	-		-		-		-
Unconditioned transplant		4	25.0%	-	-	2	(25.0%)	2	(18.1%)	0.384
Alemtuzumab conditioning only		-	-	2	(15.4%)	1	(11.1%)	-	-	0.144
**Post-HSCT complications**										
VOD		-	-	1	(6.7%)	1	(11.1%)	-	-	0.431
TMA		1	(6.3%)	1	(6.7%)	1	(11.1%)	1	(9.1%)	1.000
PN support		9	(56.3%)	6	(40.0%)	4	(44.4%)	5	(45.5%)	0.860
Intensive care admission		3	(18.8%)	6	(40.0%)	2	(22.2%)	3	(27.3%)	0.840
**Post-HSCT infections**										
Any viremia		7	(43.8%)	3	(20.0%)	2	(22.2%)	1	(9.1%)	0.237
CMV viremia		-	-	3	(20.0%)	2	(22.2%)	-	-	0.078
Adenoviremia		6	(37.5%)	2	(13.3%)	-	-	1	(9.1%)	0.113
EBV viremia		1	(6.3%)	-	-	1	(11.1%)	-	-	0.544
HHV6 viremia		1	(6.3%)	-	-	-	-	-	-	1.000
Respiratory viral infection		6	(37.5%)	2	(13.3%)	3	(33.3%)	3	(27.3%)	0.484
Gastrointestinal viral infection		3	(18.8%)	-	-	2	(22.2%)	-	-	0.103
Fungal infection		-	-	-	-	2	(22.2%)	-	-	0.021*

Categorical variables analyzed using Fisher’s exact test. Non-parametric variables analyzed using independent-samples Kruskal–Wallis test. Survival curves analyzed using log-rank test

^*^Reaches significance at *p* < 0.05

^**^Excluding patients with ADA SCID, who received pegylated ADA while awaiting MUD search, if MFD were unavailable

**Fig. 1:**
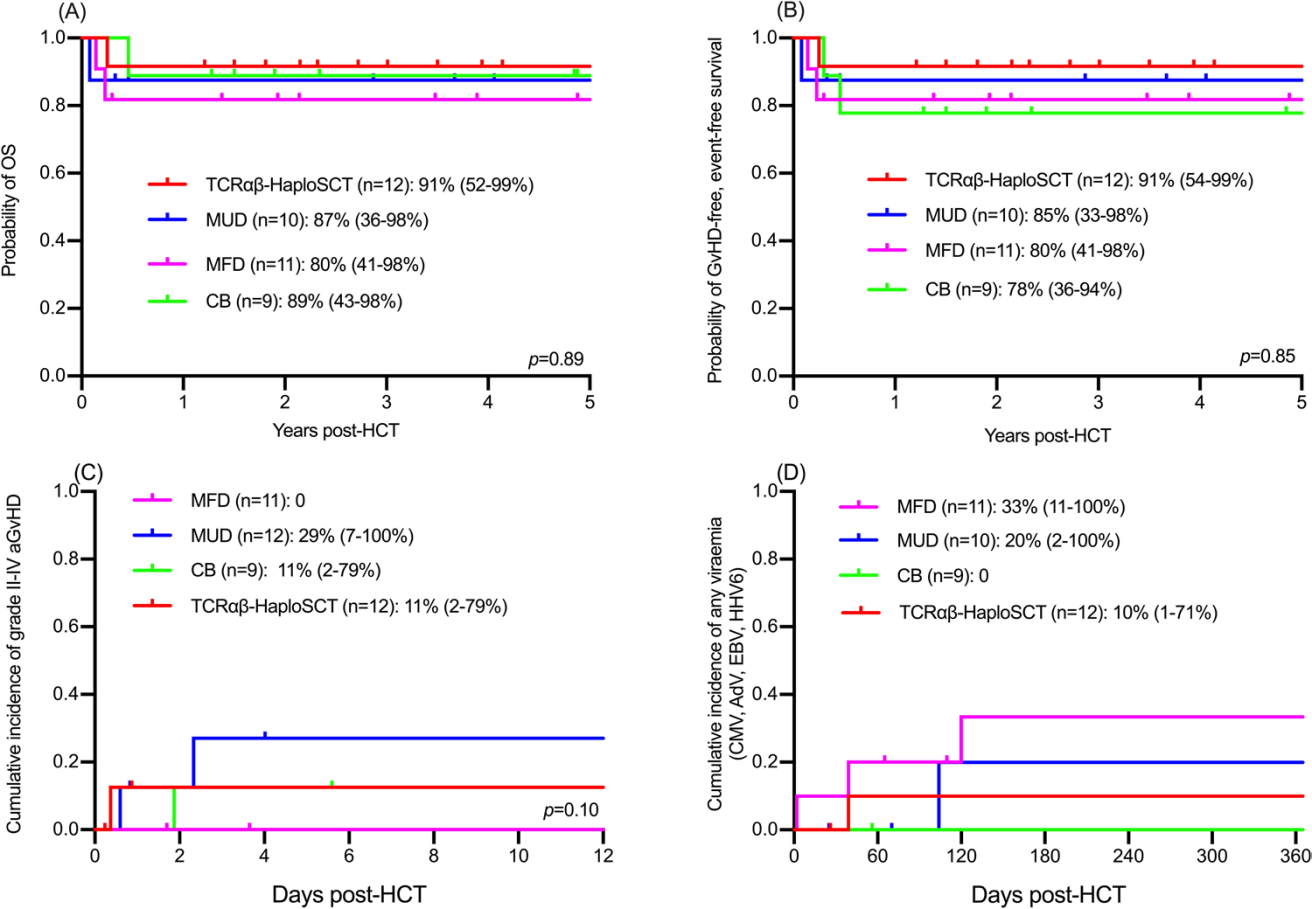
Overall survival (OS, **a**) and grade II–IV acute or chronic GvHD- and event-free survival (GEFS, **b**) at 3 years post-HSCT, cumulative incidence of grade II–IV acute GvHD (**c**), and cumulative incidence of any viremia in the first year post-HSCT (**d**) for conditioned transplants according to donor type. Numerical values represent probability % (95% confidence interval). Probabilities calculated using log-rank test for **a** and **b**, and Gray’s test for **c** and **d**. Events were defined as death or second procedure

The median follow-up duration of surviving patients was 3.5 years post-HSCT (range: 0.3–8.4 years) at the point of data collection. Three-year overall survival (OS) for the entire cohort is 88% (95% confidence interval: 74–94%). For conditioned transplants (*n* = 41), the 3-year OS was 91% (52–99%) for TCRαβ-HaploSCT, 80% (41–98%) for MFD, 87% (36–98%) for MUD, and 89% (43–89%) for CB (*p* = 0.89, **Fig. ****1a**). Graft-versus-host disease (GvHD)–free/event-free survival (GEFS, defined as survival without grade II–IV acute GvHD, chronic GvHD, or second procedure) at 3 years post-HSCT was 91% (52–99%) for TCRαβ-HaploSCT, 80% (41–98%) for MFD, 85% (33–98%) for MUD, and 78% (36–94%) for CB (*p* = 0.85, **Fig. ****1b**).

Subgroups did not differ in post-HSCT complications (**Table ****1**). Cumulative incidence of grade II–IV acute GvHD was 11% (2–79%) after TCRαβ-HaploSCT, 0 after MFD, 29% (7–100%) after MUD, and 11% (2–79%) after CB (*p* = 0.10, **Fig. ****1c**). Cumulative incidence of any viremia from CMV, adenovirus, EBV, or HHV6 over the first year post-HSCT was 10% (1–71%) for TCRαβ-HaploSCT, 33% (11–100%) for MFD, 20% (2–100%) for MUD, and 0 for CB recipients (*p* = 0.29, **Fig. ****1d**). There was no significant difference in incidence of respiratory (*p* = 0.484) or gastrointestinal viral infection (*p* = 0.103). Post-HSCT fungal infection occurred in two patients, both MUD recipients. All patients engrafted, with one patient requiring second HSCT due to severe grade IV acute GvHD after CB transplant.

**Fig. 2:**
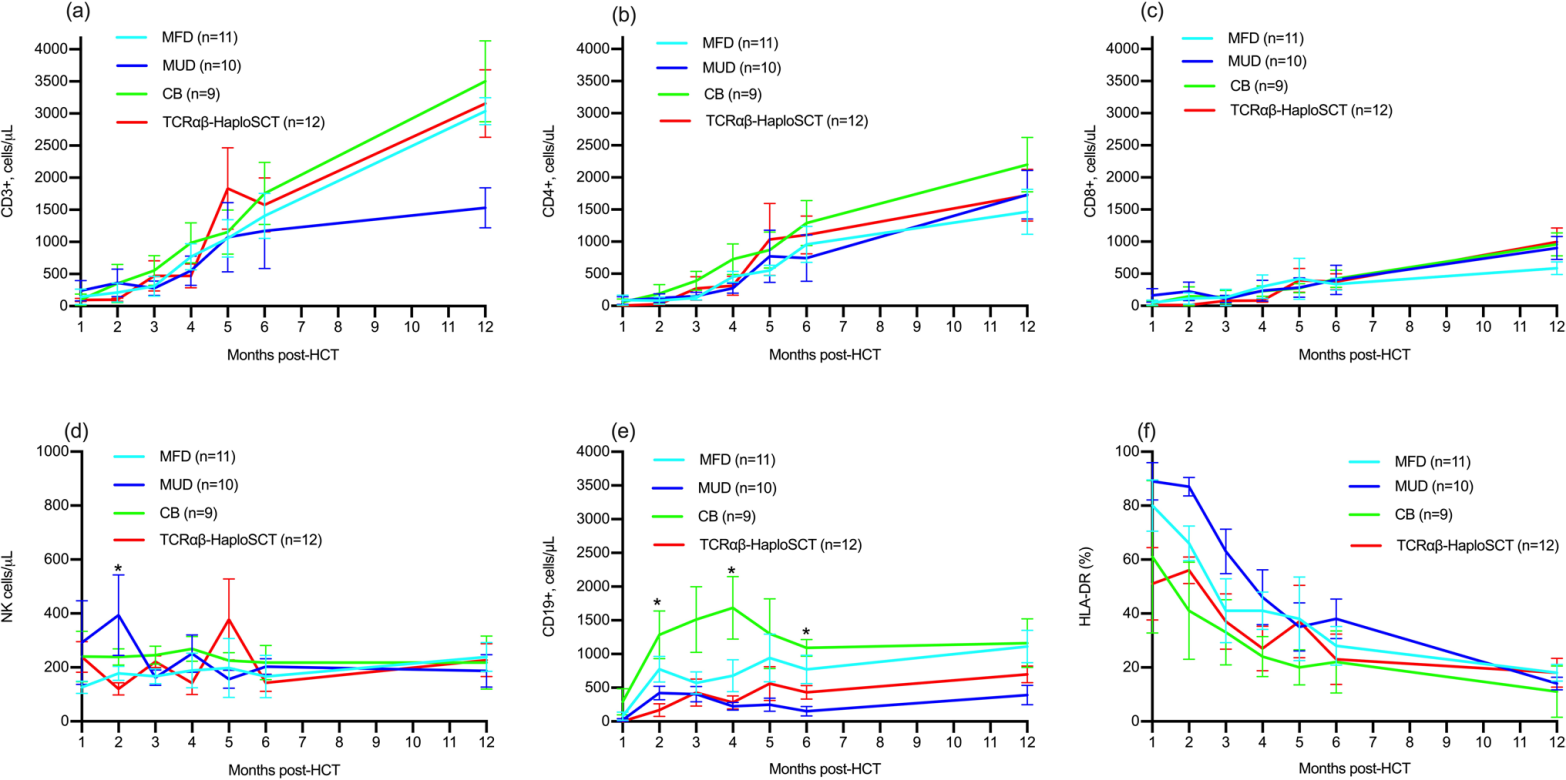
Kinetics of lymphocyte reconstitution for CD3 + (**a**), CD4 + (**b**), CD8 + (**c**), NK (**d**), and CD19 + (**e**) lymphocytes, and proportion of activated lymphocytes (**F**) represented by HLA-DR positivity, for conditioned transplants according to donor type. Bars represent one standard error margin. Mean values ± SD are available for CD3 + , CD4 + , CD19 + , and NK cells in Supplemental Table S2 in the Online Repository. *Significant at *p* < 0.05

CD3 + lymphocyte counts were lowest after TCRαβ-HaploSCT at first month (median: 76 cells/μL, range: 9–283, *p* = 0.333) and second month post-HSCT (mean: 74.5 cells/μL, range: 16–321, *p* = 0.949) compared to other conditioned donor types (**Fig. ****2a**). Detailed lymphocyte reconstitution kinetics over the first year post-HSCT by conditioned donor type are summarized in Fig. 2 and Supplemental Table S2. CD4 + and CD8 + lymphocyte reconstitution over the first year post-HSCT was comparable between donor types (**Fig. **2b and c). At month 6 post-HSCT, 62.1% of all patients with data available had a CD4 + count > 500 cells/μL (TCRαβ/CD19: 70.0%; others: 57.9%, *p* = 0.768). By month 3 post-HSCT, 40.5% of all patients had detectable naïve CD4 + lymphocytes (TCRαβ-HaploSCT: 45.5%). CD19 + lymphocyte recovered significantly quicker in CB graft recipients at month 2 (*p* < 0.001), month 4 (*p* = 0.008), and month 6 (*p* = 0.004) compared to other donor sources (**Fig. ****2e**). Both conditioned TCRαβ-HaploSCT and T-replete grafts maintained similar donor chimerism at 12 months post-HSCT in whole blood (TCRαβ-HaploSCT, all 100%; T-replete graft median chimerism: 100%, range 70–100%) with comparable rates of freedom from immunoglobulin replacement in patients followed beyond 1 year post-HSCT (TCRαβ-HaploSCT, 7/10; T-replete graft, 16/19 [70% vs 84.2%, *p* = 0.331]).

**Fig. 3:**
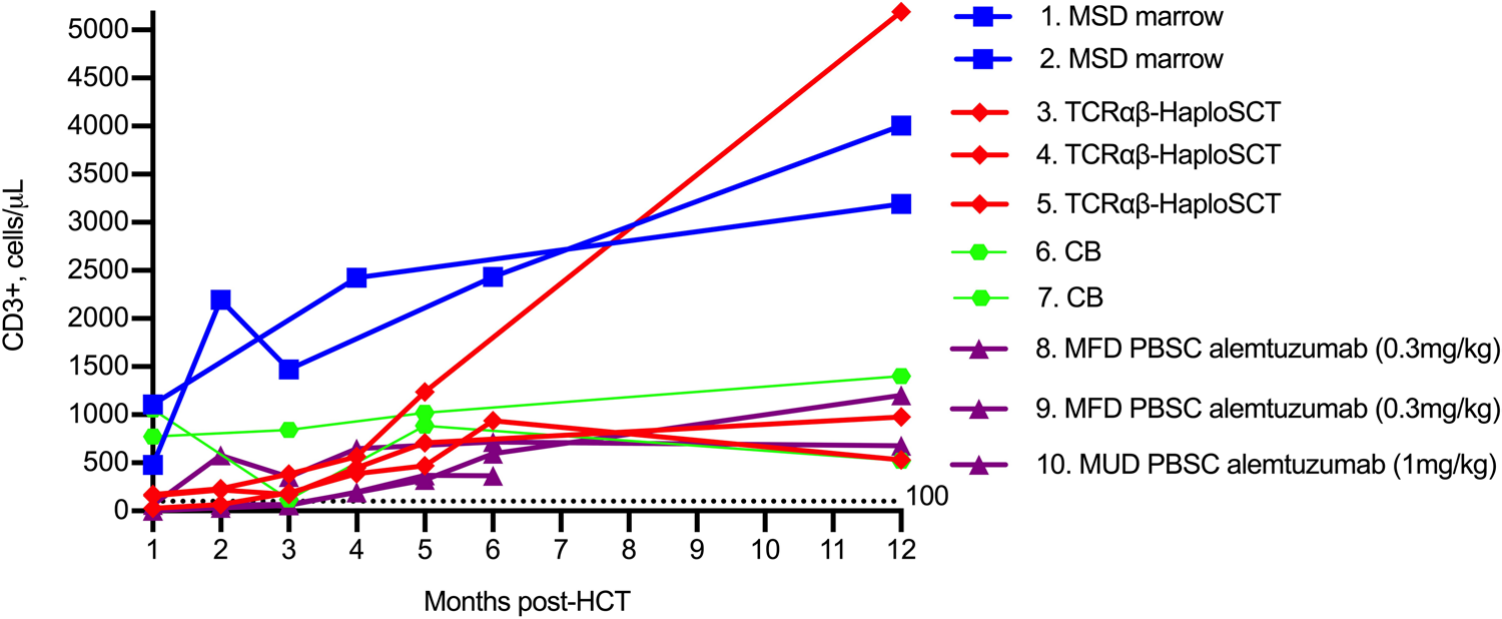
Kinetics of CD3 + lymphocyte reconstitution for unconditioned and serotherapy-only transplants over the first year post-HSCT by donor source

**Fig. 4:**
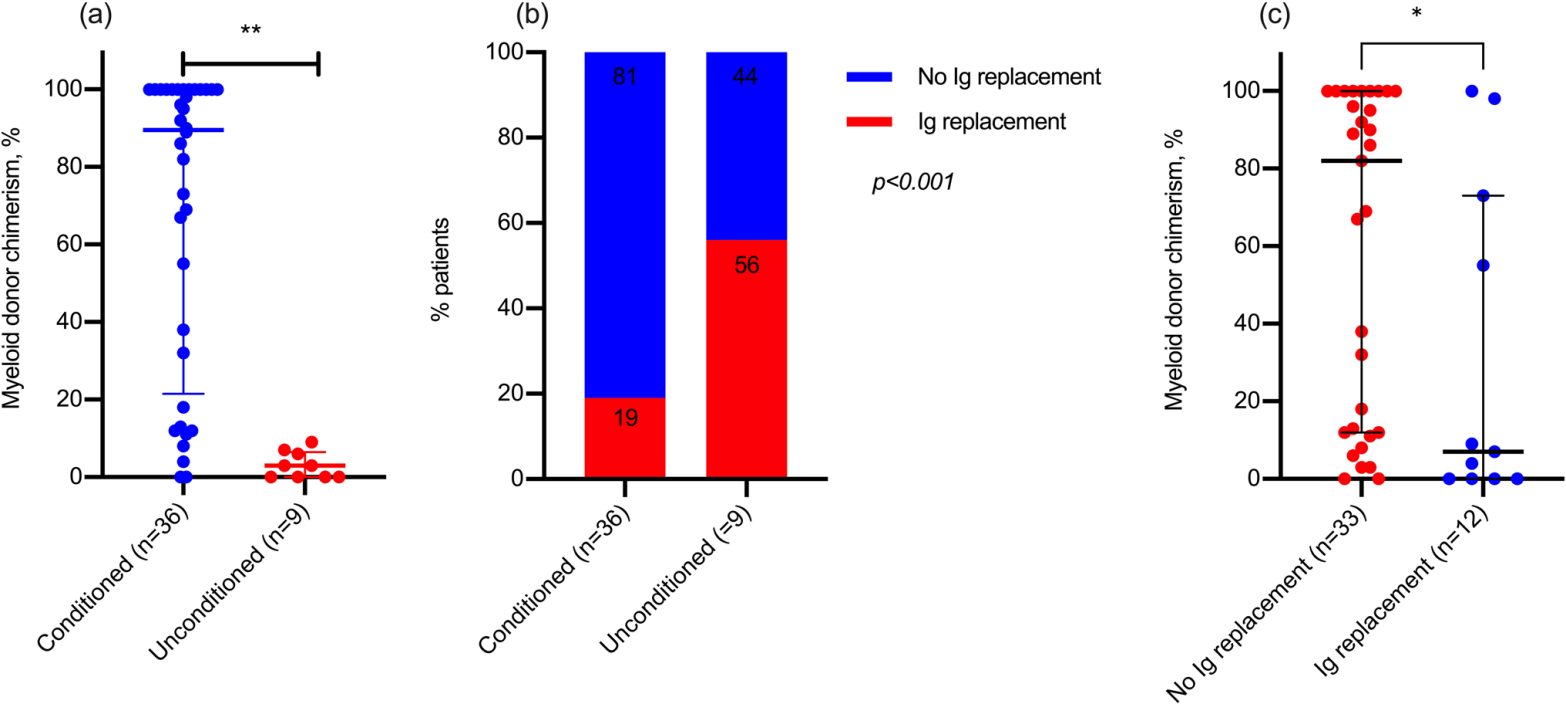
Donor myeloid chimerism (**a**) and requirement for immunoglobulin (Ig) replacement at latest-follow-up (**b**) by conditioned or unconditioned transplant, and donor myeloid chimerism by immunoglobulin dependence (**c**) for all patients at latest follow-up. Patients receiving alemtuzumab-only have been classified as “unconditioned.” *Significant at *p* < 0.05. **Significant at *p* < 0.01

Patients who received unconditioned transplants (*n* = 10) were sicker at the point of transplant, with higher rates of BCGosis (35.7% vs 8.8% for conditioned, *p* = 0.01), pre-HSCT lung disease (71.4% vs 32.4%, *p* = 0.024), and fungal infection (28.6% vs 5.9%, *p* = 0.052). For unconditioned or alemtuzumab-only transplant recipients, CD3 + recovery was faster following unconditioned MSD marrow grafts (**Fig. ****3**). Unconditioned infusions did not differ to conditioned transplants in incidence of post-HSCT intensive care admission (37.5% vs 16.3% for conditioned, *p* = 0.165), mortality (12.5% vs 11.6%), or any viremia post-HSCT (25.0% vs 25.6%). Unconditioned infusions resulted in poorer myeloid chimerism (median 3%, range: 0–9% vs 47%, range: 0–100%, *p* < 0.001) and higher rates of immunoglobulin replacement at latest follow-up (60.0% vs 12.0%, *p* < 0.001; **Fig. ****4a**** and **b). Across all patients, higher myeloid chimerism correlated with freedom from immunoglobulin replacement (**Fig. ****4c**).

Overall, 6 patients (11.8%) died, five following conditioned transplants. One patient with DNA ligase IV-SCID died following TCRαβ-HaploSCT from disseminated adenovirus; one patient with JAK3-SCID died following an MFD transplant due to disseminated CMV with TMA; three patients with Artemis SCID died after one MFD transplant (respiratory failure from RSV pneumonitis) and two MUD transplants (multi-organ failure with influenza A in one, and pulmonary TMA in the other). Deaths occurred at a median of 83 days post-HSCT (range: 28–167 days). One patient with Artemis-SCID who received an unconditioned TCRαβ/CD19-deplete haploidentical transplant while critically unwell with respiratory failure to PCP died 8 days post-HSCT.

## Discussion

The interval from diagnosis to transplant was shorter for TCRαβ-HaploSCT vs T-replete MUD when patients with ADA-SCID were excluded (**Table ****1**), confirming they are more rapidly available. TCRαβ/CD19-deplete PBSC grafts were significantly enriched with nucleated and CD34 + stem cells, and consequently myeloid engraftment occurred comparably with T-replete MSD. Reconstitution of T-lymphocytes in conditioned patients followed previous experience of TCRαβ-HaploSCT in other IEI [8, 9], with thymic output of naïve CD4 + and CD8 + lymphocytes at 3 months post-HSCT in 40.5% and 45.9% of patients, respectively. The comparatively brisk reconstitution of B-lymphocytes in recipients of CB units may reflect reduced serotherapy doses, or relatively higher proportions of B-lymphocyte precursors [5]; these cells, which do not express CD19 [13], would not account for the comparatively lower CD19 + cell dose in CB compared to MFD/MUD grafts (**Table ****1**). Both T-depleted and T-replete conditioned grafts led to similar proportions of patients with a month-6 post-HSCT CD4 + count > 500 cells/μL, with counts < 500 cells/μL previously demonstrated to be a risk factor for mortality in SCID transplant recipients [1]. Long-term, myeloid chimerism and rates of immunoglobulin use are similar between conditioned donor types. In line with the recently updated EBMT/ESID guidance [12], our data support the role of conditioning in generating higher myeloid chimerism and freedom from immunoglobulin replacement, and at our center conditioning is the default in the absence of life-threatening infection due to enhanced long-term outcomes.

Across patients undergoing HSCT for IEI, previous data from our center demonstrate improved overall and event-free survival with the advent of TCRαβ-HaploSCT compared to other methods of ex vivo T-lymphocyte depletion such as CD34 + selection, but with increased rates of viremia [11], which remain a concern due to associated morbidity and mortality [3, 9, 11]. While options for enhancing viral immunity in these patients include CD45RA + -depleted lymphocyte infusions [14], our study of SCID patients demonstrated similar cumulative incidence of any viremia post-HSCT between TCRαβ-HaploSCT and other donor types. Rates of CMV viremia were lower in our cohort compared to comparable studies looking at other inborn errors of immunity [7–9]. This may relate to our cohort being comprised solely of infants with SCID, where infection prevention measures including antiviral prophylaxis, immunoglobulin replacement, and consideration of breastfeeding cessation are universal compared to other diagnoses. Our cohort did not share the increased risk of acute GvHD demonstrated in other series [7, 8] despite comparable doses of TCRαβ + T-lymphocytes, with the cumulative incidence of clinically significant acute GvHD being comparable to other unrelated donor types. Our survival data demonstrate favorable outcomes for TCRαβ-HaploSCT recipients compared to other donor types, while the over-representation of Artemis-deficient SCID in our mortality data echoes the published experience of HSCT for this challenging genotype [1, 15].

## Conclusion

The role of TCRαβ/CD19 depletion for haploidentical donor transplant in IEI is evolving, with several series demonstrating similar survival and morbidity compared to unrelated donors [8–10], though patients with SCID have been under-represented. Our data demonstrate that conditioned TCRαβ/CD19-depleted haploidentical grafts are a safe and effective alternative for infants with SCID who lack HLA-matched family donors and support the use of conditioning pre-HSCT to improve long-term immunological recovery. We have thus amended our donor hierarchy to:

1. Matched family donor;
2. Parental TCRαβ-HaploSCT *or* matched unrelated donor,thereby removing a barrier to definitive treatment of SCID.

## Supplementary Information

Below is the link to the electronic supplementary material.

Supplementary file1 (DOCX 56 KB)

## Data Availability

Not available.

## Abbreviations

ADA: Adenosine deaminase
ATG: Anti-thymocyte globulin
CMV: Cytomegalovirus
CsA: Ciclosporin A
EBV: Epstein-Barr virus
GEFS: Acute grade II-IV graft-versus-host disease- and event-free survival
GvHD: Graft-versus-host disease
HHV6: Human herpesvirus-6
HSCT: Hematopoietic stem cell transplant
IEI: Inborn errors of immunity
MFD: Matched family donor
MMF: Mycophenolate mofetil
MSD: Matched sibling donor
MUD: Matched unrelated donor
OS: Overall survival
PBSC: Peripheral blood stem cells
PCP: *Pneumocystis **jirovecii* pneumonia
SCID: Severe combined immunodeficiency
TCRαβ: T-cell receptor α/β
TMA: Thrombotic microangiopathy
VOD: Veno-occlusive disease

## References

[1] Haddad E, Logan BR, Griffith LM, Buckley RH, Parrott RE, Prockop SE (2018). SCID genotype and 6-month posttransplant CD4 count predict survival and immune recovery. Blood.

[2] Pai S-Y, Logan BR, Griffith LM, Buckley RH, Parrott RE, Dvorak CC (2014). Transplantation outcomes for severe combined immunodeficiency, 2000–2009. N Engl J Med.

[3] Railey MD, Lokhnygina Y, Buckley RH. Long-term clinical outcome of patients with severe combined immunodeficiency who received related donor bone marrow transplants without pretransplant chemotherapy or post-transplant GVHD prophylaxis. J Pediatr [Internet]. 2009 Dec;155(6):834–840.e1. https://linkinghub.elsevier.com/retrieve/pii/S0022347609007471 10.1016/j.jpeds.2009.07.049 PMC2784223 19818451

[4] Grunebaum E, Mazzolari E, Porta F, Dallera D, Atkinson A, Reid B, et al. Bone marrow transplantation for severe combined immune deficiency. J Am Med Assoc [Internet]. 2006 Feb 1;295(5):508–18. http://www.ncbi.nlm.nih.gov/pubmed/16449616 10.1001/jama.295.5.508 16449616

[5] Elfeky R, Lazareva A, Qasim W, Veys P (2019). Immune reconstitution following hematopoietic stem cell transplantation using different stem cell sources. Expert Rev Clin Immunol [Internet].

[6] Chiesa R, Gilmour K, Qasim W, Adams S, Worth AJJ, Zhan H (2012). Omission of in vivo T-cell depletion promotes rapid expansion of naïve CD4 + cord blood lymphocytes and restores adaptive immunity within 2months after unrelated cord blood transplant. Br J Haematol.

[7] Laberko A, Bogoyavlenskaya A, Shelikhova L, Shekhovtsova Z, Balashov D, Voronin K (2017). Risk factors for and the clinical impact of cytomegalovirus and Epstein-Barr virus infections in pediatric recipients of TCR-α/β– and CD19-depleted grafts. Biol Blood Marrow Transplant [Internet].

[8] Balashov D, Shcherbina A, Maschan M, Trakhtman P, Skvortsova Y, Shelikhova L (2015). Single-center experience of unrelated and haploidentical stem cell transplantation with TCRαβ and CD19 depletion in children with primary immunodeficiency syndromes. Biol Blood Marrow Transplant [Internet].

[9] Brettig T, Smart J, Choo S, Mechinaud F, Mitchell R, Raj TS (2019). Use of TCR α+β+/CD19+–depleted haploidentical hematopoietic stem cell transplant is a viable option in patients with primary immune deficiency without matched sibling donor. J Clin Immunol.

[10] Shah RM, Elfeky R, Nademi Z, Qasim W, Amrolia P, Chiesa R (2018). T-cell receptor αβ+ and CD19+ cell–depleted haploidentical and mismatched hematopoietic stem cell transplantation in primary immune deficiency. J Allergy Clin Immunol [Internet].

[11] Lum SH, Sobh A, Carruthers K, Nademi Z, Watson H, McNaughton P (2020). Improved survival and graft function in ex vivo T-cell depleted haploidentical hematopoietic cell transplantation for primary immunodeficiency. Bone Marrow Transplant [Internet].

[12] Lankester AC, Albert MH, Booth C, Gennery AR, Güngör T, Hönig M (2021). EBMT/ESID inborn errors working party guidelines for hematopoietic stem cell transplantation for inborn errors of immunity. Bone Marrow Transplant [Internet].

[13] Wang K, Wei G, Liu D (2012). CD19: a biomarker for B cell development, lymphoma diagnosis and therapy. Exp Hematol Oncol [Internet].

[14] Brodszki N, Turkiewicz D, Toporski J, Truedsson L, Dykes J (2016). Novel treatment of severe combined immunodeficiency utilizing ex-vivo T-cell depleted haploidentical hematopoietic stem cell transplantation and CD45RA+ depleted donor lymphocyte infusions. Orphanet J Rare Dis [Internet].

[15] Cowan MJ, Gennery AR. Radiation-sensitive severe combined immunodeficiency: the arguments for and against conditioning before hematopoietic cell transplantation—what to do? J Allergy Clin Immunol [Internet]. 2015 Nov;136(5):1178–85. https://linkinghub.elsevier.com/retrieve/pii/S0091674915005898 10.1016/j.jaci.2015.04.027 PMC4641002 26055221

