# Supplementary material for: TCRαβ-Depleted Haploidentical Grafts Are a Safe Alternative to HLA-Matched Unrelated Donor Stem Cell Transplants for Infants with Severe Combined Immunodeficiency
Source: J Clin Immunol. 2022 Mar 19;42(4):851–8. doi: 10.1007/s10875-022-01239-z (PMC9166847; doi:10.1007/s10875-022-01239-z)
Supplement: Supplementary file 1 — Supplementary file1 (DOCX 56 KB) [file 10875_2022_1239_MOESM1_ESM.docx]

**Title:**

TCRαβ-depleted haploidentical grafts are a safe alternative to HLA-matched unrelated donor stem cell transplants for infants with severe combined immunodeficiency

**Authors:**

Christo Tsilifis^1,2^, Su Han Lum^1^, Zohreh Nademi^1,2^, Sophie Hambleton^1,2^, Terence J. Flood^1^, Eleri J. Williams^1^, Stephen Owens^1^, Mario Abinun^1,2^, Andrew J. Cant^1,2^, Mary A. Slatter^1,2^, Andrew R. Gennery^1,2^

^1^Paediatric Haematopoietic Stem Cell Transplant Unit, Great North Children’s Hospital (GNCH), Victoria Wing, Royal Victoria Infirmary, Newcastle upon Tyne NE1 4LP, United Kingdom;

^2^Translational and Clinical Research Institute, Faculty of Medical Sciences, Newcastle University, Newcastle upon Tyne, NE2 4HH, United Kingdom;

**Corresponding author:**

Professor Andrew R. Gennery
Clinical Resource Building, Floor 4, Block 2, Great North Children’s Hospital
Queen Victoria Road, Newcastle Upon Tyne, NE1 4LP
**Email:** a.r.gennery@ncl.ac.uk
**Telephone:** 0191 2825234
**Fax:** 0191 2730183

**Supplemental Tables**

| **Supplemental Table S1:** Expanded **b**aseline characteristics, SCID subtype, pre-HSCT comorbidities, and transplant details across donor groups. | | | | | | | | | | | | | | | | | | | |
| --- | --- | --- | --- | --- | --- | --- | --- | --- | --- | --- | --- | --- | --- | --- | --- | --- | --- | --- | --- |
|  | | TCRαβ/CD19-depleted haploidentical donor (n=16) | | | | HLA-matched T-replete graft | | | | | | | | | | | Total | *p*-value | |
|  |  |  |  |  |  | Family donor (n=15) | | Adult unrelated donor (n=9) | | | | Cord blood donor (n=11) | | | | |  |  |  |
| **Demographics** | | | | | | | | | | | | | | | | | | | |
| Newborn diagnosis | | 4 | (25.0%) |  |  | 6 | (40.0%) |  |  | 2 | (22.2%) |  |  | 2 | (18.2%) |  | 14 |  | 0.681 |
| Male sex | | 4 | (25.0%) |  |  | 4 | (26.7%) |  |  | 3 | (33.3%) |  |  | 4 | (36.4%) |  | 15 |  | 0.904 |
| Phenotype | T-B+NK+ | 4 | (25.0%) |  |  | 2 | (13.3%) |  |  | 1 | (11.1%) |  |  | 1 | (9.1%) |  | 8 |  | 0.517 |
|  | T-B+NK- | 6 | (37.5%) |  |  | 5 | (33.3%) |  |  | 1 | (11.1%) |  |  | 2 | (18.2%) |  | 14 |  |  |
|  | T-B-NK+ | 5 | (31.3%) |  |  | 4 | (26.7%) |  |  | 4 | (44.4%) |  |  | 3 | (27.3%) |  | 16 |  |  |
|  | T-B-NK- | 1 | (6.3%) |  |  | 4 | (26.7%) |  |  | 3 | (33.3%) |  |  | 5 | (45.5%) |  | 13 |  |  |
| Diagnosis | Familial Omenn-like syndrome | - | - |  |  | 1 | (6.7%) |  |  | - | - |  |  | - | - |  | 1 |  | 0.233 |
|  | SCID: ADA | - | - |  |  | 4 | (26.7%) |  |  | 3 | (33.3%) |  |  | 5 | (45.5%) |  | 12 |  |  |
|  | SCID: Artemis | 3 | (18.8%) |  |  | 2 | (13.3%) |  |  | 2 | (22.2%) |  |  | 1 | (9.1%) |  | 8 |  |  |
|  | SCID: common gamma chain | 5 | (31.3%) |  |  | 1 | (6.7%) |  |  | 1 | (11.1%) |  |  | 2 | (18.2%) |  | 9 |  |  |
|  | SCID: DNA ligase IV | 1 | (6.3%) |  |  | - | - |  |  | - | - |  |  | - | - |  | 1 |  |  |
|  | SCID: IL7RA | 2 | (12.5%) |  |  | 1 | (6.7%) |  |  | 1 | (11.1%) |  |  | 1 | (9.1%) |  | 5 |  |  |
|  | SCID: JAK3 | 1 | (6.3%) |  |  | 4 | (26.7%) |  |  | - | - |  |  | - | - |  | 5 |  |  |
|  | SCID: RAG1 | 1 | (6.3%) |  |  | 2 | (13.3%) |  |  | 2 | (22.2%) |  |  | 1 | (9.1%) |  | 6 |  |  |
|  | SCID: RAG2 | - | - |  |  | - | - |  |  | - | - |  |  | 1 | (9.1%) |  | 1 |  |  |
|  | SCID: Reticular dysgenesis | 1 | (6.3%) |  |  | - | - |  |  | - | - |  |  | - | - |  | 1 |  |  |
|  | SCID: T_low_B+NK+ | 2 | (12.5%) |  |  | - | - |  |  | - | - |  |  | - | - |  | 2 |  |  |
| **Pre-HSCT comorbidities** | | | | | | | | | | | | | | | | | | | |
| History of viremia pre-HSCT | | 4 | (25.0%) |  |  | 4 | (26.7%) |  |  | 2 | (22.2%) |  |  | - | - |  | 10 |  | 0.292 |
| CMV viremia at HSCT | | - | - |  |  | 2 | (13.3%) |  |  | 2 | (22.2%) |  |  | - | - |  | 4 |  | 0.078 |
| Adenoviremia at HSCT | | 2 | (12.5%) |  |  | - | - |  |  | - | - |  |  | - | - |  | 2 |  | 0.325 |
| HHV6 viremia at HSCT | | 1 | (6.3%) |  |  | - | - |  |  | - | - |  |  | - | - |  | 1 |  | 1.000 |
| Probable or confirmed fungal infection | | 2 | (12.5%) |  |  | 1 | (6.7%) |  |  | 2 | (22.2%) |  |  | 1 | (9.1%) |  | 6 |  | 0.749 |
| Pneumocystis infection | | 5 | (31.3%) |  |  | 2 | (13.3%) |  |  | 1 | (11.1%) |  |  | 2 | (18.2%) |  | 10 |  | 0.592 |
| BCG infection | Disseminated | 2 | (12.5%) |  |  | 1 | (6.7%) |  |  | 1 | (11.1%) |  |  | - | - |  | 4 |  | 0.252 |
|  | Superficial | 2 | (12.5%) |  |  | - | - |  |  | 2 | (22.2%) |  |  | - | - |  | 4 |  |  |
| Respiratory viral infection | | 7 | (43.8%) |  |  | 6 | (40.0%) |  |  | 5 | (55.6%) |  |  | 3 | (27.3%) |  | 21 |  | 0.654 |
| Gastrointestinal viral infection | | 9 | (56.3%) |  |  | 4 | (26.7%) |  |  | 3 | (33.3%) |  |  | 4 | (36.4%) |  | 20 |  | 0.392 |
| PN dependence | | 4 | (25.0%) |  |  | 2 | (14.3%) |  |  | 1 | (11.1%) |  |  | 2 | (18.2%) |  | 9 |  | 0.885 |
| Growth failure | | 7 | (43.8%) |  |  | 7 | (46.7%) |  |  | 2 | (22.2%) |  |  | 5 | (45.5%) |  | 21 |  | 0.674 |
| Admission to NICU‎/PICU | | 4 | (25.0%) |  |  | 3 | (20.0%) |  |  | 2 | (22.2%) |  |  | 4 | (36.4%) |  | 13 |  | 0.805 |
| **Transplant characteristics** | | | | | | | | | | | | | | | | | | | |
| Stem cell source | Cord blood | - | - |  |  | - | - |  |  | - | - |  |  | 11 | (100.0%) |  | *<0.001** | | |
|  | Marrow | - | - |  |  | 10 | (66.7%) |  |  | 3 | (33.3%) |  |  | - | - |  |  |  |  |
|  | PBSC | 16 | (100.0%) |  |  | 5 | (33.3%) |  |  | 6 | (66.7%) |  |  | - | - |  |  |  |  |
| Serotherapy used | None | 4 | (25.0%) |  |  | 2 | (13.3%) |  |  | - | - |  |  | 2 | (18.2%) |  | *<0.001** | | |
|  | Alemtuzumab | - | - |  |  | 13 | (86.7%) |  |  | 9 | (100.0%) |  |  | 8 | (72.7%) |  |  |  |  |
|  | ATG (Grafalon) | 12 | (75.0%) |  |  | - | - |  |  | - | - |  |  | - | - |  |  |  |  |
|  | ATG (Thymoglobulin) | - | - |  |  | - | - |  |  | - | - |  |  | 1 | (9.1%) |  |  |  |  |
| GvHD prophylaxis | None | 9 | (56.3%) |  |  | - | - |  |  | - | - |  |  | - | - |  | *<0.001** | | |
|  | CsA only | 2 | (12.5%) |  |  | 1 | (6.7%) |  |  | - | - |  |  | - | - |  |  |  |  |
|  | CsA + MMF | 5 | (31.3%) |  |  | 14 | (93.3%) |  |  | 9 | (100.0%) |  |  | 11 | (100.0%) |  |  |  |  |
| CD45RO+ add-back procedure | | 3 | (18.8%) |  |  | 1 | (6.7%) |  |  | - | - |  |  | - | - |  | 0.399 | | |
| Growth failure defined as weight <9th centile at time of HSCT. Categorical variables analysed using Fisher’s exact test. Non-parametric variables analysed using independent-samples Kruskal-Wallis test.  ns: not statistically significant at p<0.05  * indicates p<0.05. | | | | | | | | | | | | | | | | | | | |
|  | | | | | | | | | | | | | | | | | | | |

**Supplemental Table S2:** Lymphocyte reconstitution kinetics of CD3+, CD4+, CD19+ and NK cells over the first 12 months post-HSCT for conditioned HSCT, expressed as mean cell count ± standard error (SE).

* indicates *p*<0.05.

|  | | CD3+ count (cells‎/microlitre) | | | | | | | | | | | | | | | | | | | | |
| --- | --- | --- | --- | --- | --- | --- | --- | --- | --- | --- | --- | --- | --- | --- | --- | --- | --- | --- | --- | --- | --- | --- |
|  |  | Months post-HSCT | | | | | | | | | | | | | | | | | | | | |
|  |  | **1** | | | **2** | | | **3** | | | **4** | | | **5** | | | **6** | | | **12** | | |
|  |  | Mean | ±SE | n | Mean | ±SE | n | Mean | ±SE | n | Mean | ±SE | n | Mean | ±SE | n | Mean | ±SE | n | Mean | ±SE | n |
| Donor type | T-replete matched family (n=11) | 146 | 119 | 9 | 213 | 147 | 9 | 310 | 156 | 6 | 768 | 203 | 7 | 1055 | 290 | 4 | 1405 | 351 | 7 | 3037 | 210 | 5 |
|  | T-replete adult MUD (n=10) | 240 | 157 | 8 | 361 | 214 | 9 | 278 | 113 | 9 | 550 | 229 | 8 | 1073 | 538 | 6 | 1170 | 583 | 6 | 1530 | 313 | 7 |
|  | T-replete unrelated cord blood (n=9) | 101 | 90 | 8 | 353 | 298 | 7 | 556 | 231 | 7 | 985 | 311 | 8 | 1154 | 343 | 6 | 1756 | 483 | 4 | 3501 | 631 | 7 |
|  | TCRαβ/CD19-Haplo(n=12) | 97 | 26 | 10 | 98 | 25 | 12 | 470 | 235 | 8 | 470 | 184 | 7 | 1832 | 632 | 6 | 1576 | 419 | 9 | 3154 | 525 | 9 |
|  | *p*-value | 0.10 | | | 0.94 | | | 0.71 | | | 0.90 | | | 0.68 | | | 0.56 | | | 0.23 | | |

|  | | CD4+ count (cells‎/microlitre) | | | | | | | | | | | | | | | | | | | | |
| --- | --- | --- | --- | --- | --- | --- | --- | --- | --- | --- | --- | --- | --- | --- | --- | --- | --- | --- | --- | --- | --- | --- |
|  |  | Months post-HSCT | | | | | | | | | | | | | | | | | | | | |
|  |  | **1** | | | **2** | | | **3** | | | **4** | | | **5** | | | **6** | | | **12** | | |
|  |  | Mean | ±SE | n | Mean | ±SE | n | Mean | ±SE | n | Mean | ±SE | n | Mean | ±SE | n | Mean | ±SE | n | Mean | ±SE | n |
| Donor type | T-replete matched family (n=11) | 89 | 76 | 9 | 82 | 57 | 9 | 125 | 33 | 6 | 454 | 85 | 7 | 552 | 82 | 4 | 958 | 282 | 7 | 1465 | 350 | 10 |
|  | T-replete adult MUD (n=10) | 82 | 59 | 8 | 120 | 68 | 9 | 151 | 60 | 9 | 276 | 77 | 8 | 771 | 408 | 6 | 745 | 362 | 6 | 1732 | 379 | 9 |
|  | T-replete unrelated cord blood (n=9) | 59 | 51 | 8 | 189 | 143 | 7 | 390 | 148 | 7 | 727 | 240 | 8 | 871 | 280 | 6 | 1291 | 350 | 4 | 2199 | 425 | 5 |
|  | TCRαβ/CD19-Haplo(n=12) | 6 | 3 | 10 | 25 | 8 | 12 | 271 | 183 | 8 | 314 | 149 | 7 | 1033 | 562 | 6 | 1105 | 293 | 9 | 1723 | 403 | 9 |
|  | *p*-value | 0.58 | | | 0.58 | | | 0.48 | | | 0.19 | | | 0.57 | | | 0.48 | | | 0.47 | | |

|  | | CD8+ count (cells‎/microlitre) | | | | | | | | | | | | | | | | | | | | |
| --- | --- | --- | --- | --- | --- | --- | --- | --- | --- | --- | --- | --- | --- | --- | --- | --- | --- | --- | --- | --- | --- | --- |
|  |  | Months post-HSCT | | | | | | | | | | | | | | | | | | | | |
|  |  | **1** | | | **2** | | | **3** | | | **4** | | | **5** | | | **6** | | | **12** | | |
|  |  | Mean | ±SE | n | Mean | ±SE | n | Mean | ±SE | n | Mean | ±SE | n | Mean | ±SE | n | Mean | ±SE | n | Mean | ±SE | n |
| Donor type | T-replete matched family (n=11) | 50 | 39 | 9 | 113 | 87 | 9 | 132 | 126 | 6 | 299 | 183 | 7 | 422 | 318 | 4 | 337 | 90 | 7 | 585 | 101 | 10 |
|  | T-replete adult MUD (n=10) | 161 | 143 | 9 | 226 | 143 | 9 | 105 | 57 | 9 | 236 | 161 | 8 | 286 | 152 | 6 | 403 | 230 | 6 | 901 | 180 | 9 |
|  | T-replete unrelated cord blood (n=9) | 41 | 39 | 8 | 150 | 150 | 7 | 139 | 102 | 7 | 228 | 82 | 8 | 274 | 71 | 6 | 423 | 135 | 4 | 957 | 178 | 4 |
|  | TCRαβ/CD19-Haplo(n=12) | 12 | 4 | 10 | 11 | 3 | 12 | 79 | 36 | 8 | 81 | 27 | 7 | 398 | 188 | 6 | 375 | 122 | 9 | 995 | 217 | 9 |
|  | *p*-value | 0.91 | | | 0.47 | | | 0.95 | | | 0.22 | | | 0.66 | | | 0.72 | | | 0.47 | | |

|  | | NK+ count (cells‎/microlitre) | | | | | | | | | | | | | | | | | | | | |
| --- | --- | --- | --- | --- | --- | --- | --- | --- | --- | --- | --- | --- | --- | --- | --- | --- | --- | --- | --- | --- | --- | --- |
|  |  | Months post-HSCT | | | | | | | | | | | | | | | | | | | | |
|  |  | **1** | | | **2** | | | **3** | | | **4** | | | **5** | | | **6** | | | **12** | | |
|  |  | Mean | ±SE | n | Mean | ±SE | n | Mean | ±SE | n | Mean | ±SE | n | Mean | ±SE | n | Mean | ±SE | n | Mean | ±SE | n |
| Donor type | T-replete matched family (n=11) | 126 | 22 | 9 | 178 | 26 | 10 | 167 | 27 | 6 | 189 | 64 | 7 | 198 | 109 | 4 | 166 | 78 | 7 | 239 | 53 | 10 |
|  | T-replete adult MUD (n=10) | 292 | 155 | 8 | 394 | 150 | 9 | 162 | 28 | 9 | 251 | 69 | 8 | 156 | 34 | 6 | 203 | 29 | 6 | 198 | 60 | 9 |
|  | T-replete unrelated cord blood (n=9) | 240 | 93 | 8 | 239 | 30 | 7 | 245 | 34 | 7 | 269 | 46 | 8 | 225 | 29 | 6 | 218 | 64 | 4 | 218 | 99 | 4 |
|  | TCRαβ/CD19-Haplo(n=12) | 239 | 93 | 10 | 120 | 22 | 12 | 221 | 22 | 8 | 142 | 43 | 7 | 378 | 150 | 6 | 143 | 32 | 9 | 227 | 61 | 9 |
|  | *p*-value | 0.36 | | | 0.03 | | | 0.10 | | | 0.39 | | | 0.52 | | | 0.46 | | | 0.85 | | |

|  | | CD19+ count (cells‎/microlitre) | | | | | | | | | | | | | | | | | | | | |
| --- | --- | --- | --- | --- | --- | --- | --- | --- | --- | --- | --- | --- | --- | --- | --- | --- | --- | --- | --- | --- | --- | --- |
|  |  | Months post-HSCT | | | | | | | | | | | | | | | | | | | | |
|  |  | **1** | | | **2** | | | **3** | | | **4** | | | **5** | | | **6** | | | **12** | | |
|  |  | Mean | ±SE | n | Mean | ±SE | n | Mean | ±SE | n | Mean | ±SE | n | Mean | ±SE | n | Mean | ±SE | n | Mean | ±SE | n |
| Donor type | T-replete matched family (n=11) | 78 | 62 | 9 | 775 | 190 | 10 | 572 | 163 | 6 | 679 | 239 | 7 | 943 | 356 | 4 | 773 | 211 | 7 | 1111 | 242 | 10 |
|  | T-replete adult MUD (n=10) | 23 | 21 | 8 | 423 | 101 | 9 | 406 | 113 | 9 | 226 | 59 | 8 | 248 | 98 | 6 | 153 | 70 | 6 | 393 | 145 | 9 |
|  | T-replete unrelated cord blood (n=9) | 291 | 194 | 8 | 1284 | 353 | 7 | 1512 | 486 | 7 | 1686 | 465 | 8 | 1300 | 520 | 6 | 1093 | 125 | 4 | 1163 | 362 | 4 |
|  | TCRαβ/CD19-Haplo(n=12) | 2 | 2 | 10 | 167 | 93 | 12 | 429 | 199 | 8 | 283 | 98 | 7 | 562 | 251 | 6 | 430 | 102 | 9 | 699 | 123 | 9 |
|  | *p*-value | 0.07 | | | <0.001* | | | 0.07 | | | 0.008* | | | 0.13 | | | 0.004* | | | 0.09 | | |

|  | | HLA-DR (%) | | | | | | | | | | | | | | | | | | | | |
| --- | --- | --- | --- | --- | --- | --- | --- | --- | --- | --- | --- | --- | --- | --- | --- | --- | --- | --- | --- | --- | --- | --- |
|  |  | Months post-HSCT | | | | | | | | | | | | | | | | | | | | |
|  |  | **1** | | | **2** | | | **3** | | | **4** | | | **5** | | | **6** | | | **12** | | |
|  |  | Mean | ±SE | n | Mean | ±SE | n | Mean | ±SE | n | Mean | ±SE | n | Mean | ±SE | n | Mean | ±SE | n | Mean | ±SE | n |
| Donor type | T-replete matched family (n=11) | 80 | 10 | 4 | 66 | 6 | 7 | 41 | 12 | 6 | 41 | 7 | 6 | 38 | 16 | 4 | 28 | 7 | 7 | 18 | 3 | 10 |
|  | T-replete adult MUD (n=10) | 89 | 7 | 3 | 87 | 3 | 3 | 63 | 8 | 7 | 46 | 10 | 7 | 35 | 9 | 6 | 38 | 7 | 6 | 14 | 2 | 9 |
|  | T-replete unrelated cord blood (n=9) | 61 | 28 | 2 | 41 | 18 | 4 | 33 | 12 | 7 | 24 | 7 | 8 | 20 | 7 | 6 | 22 | 12 | 4 | 11 | 10 | 4 |
|  | TCRαβ/CD19-Haplo(n=12) | 51 | 13 | 6 | 56 | 5 | 8 | 37 | 10 | 8 | 27 | 8 | 7 | 37 | 7 | 6 | 23 | 9 | 9 | 18 | 5 | 9 |
|  | *p*-value | 0.18 | | | 0.05 | | | 0.07 | | | 0.16 | | | 0.56 | | | 0.36 | | | 0.49 | | |
